# Identifying the etiology and pathophysiology underlying stunting and environmental enteropathy: study protocol of the AFRIBIOTA project

**DOI:** 10.1186/s12887-018-1189-5

**Published:** 2018-07-19

**Authors:** Pascale Vonaesch, Rindra Randremanana, Jean-Chrysostome Gody, Jean-Marc Collard, Tamara Giles-Vernick, Maria Doria, Inès Vigan-Womas, Pierre-Alain Rubbo, Aurélie Etienne, Emilson Jean Andriatahirintsoa, Nathalie Kapel, Eric Brown, Kelsey E. Huus, Darragh Duffy, B.Brett Finlay, Milena Hasan, Francis Allen Hunald, Annick Robinson, Alexandre Manirakiza, Laura Wegener-Parfrey, Muriel Vray, Philippe J. Sansonetti, for the AFRIBIOTA Investigators, Emilson Jean Andriatahirintsoa, Laurence Barbot-Trystram, Robert Barouki, Alexandra Bastaraud, Jean-Marc Collard, Maria Doria, Darragh Duffy, Aurélie Etienne, B. Brett Finlay, Serge Ghislain Djorie, Tamara Giles-Vernick, Bolmbaye Privat Gondje, Jean-Chrysostome Gody, Milena Hasan, Jean-Michel Héraud, François Huetz, Francis Allan Hunald, Nathalie Kapel, Jean-Pierre Lombart, Alexandre Manirakiza, Synthia Nazita Nigatoloum, Sophie Novault, Laura Wegener Parfrey, Lisette Raharimalala, Maheninasy Rakotondrainipiana, Rindra Randremanana, Harifetra Mamy Richard Randriamizao, Frédérique Randrianirina, Annick Robinson, Pierre-Alain Rubbo, Philippe Sansonetti, Laura Schaeffer, Ionela Gouandjika-Vassilache, Pascale Vonaesch, Sonia Sandrine Vondo, Inès Vigan-Womas

**Affiliations:** 1 Unité de Pathogénie Microbienne Moléculaire, Institut Pasteur, 28 Rue du Dr. Roux, 75015 Paris, France; 2 Unité d’Epidémiologie et de Recherche Clinique, Institut Pasteur de Madagascar, BP 1274 Ambatofotsikely, Avaradoha, 101 Antananarivo, Madagascar; 3 Centre Pédiatrique de Bangui, Avenue de l’Indépendance, Bangui, Central African Republic; 4 Unité de Bactériologie Expérimentale, Institut Pasteur de Madagascar, BP 1274 Ambatofotsikely, Avaradoha, 101 Antananarivo, Madagascar; 5 Unité d’Epidémiologie des Maladies Emergentes, Institut Pasteur, 28 Rue du Dr. Roux, 75015 Paris, France; 6 Unité d’Immunologie des Maladies Infectieuses, Institut Pasteur de Madagascar, BP 1274 Ambatofotsikely, Avaradoha, 101 Antananarivo, Madagascar; 7 Laboratoire d’Analyses Médicales, Institut Pasteur de Bangui, Avenue de l’Indépendance, Bangui, Central African Republic; 8 Centre Hospitalier Universitaire Mère-Enfant de Tsaralalàna (CHUMET), rue Patrice Lumumba, Tsaralalàna, 101 Antananarivo, Madagascar; 9 Laboratoire de Coprologie Fonctionnelle, Hôpital Pitié-Salpêtrière, 47-83 Bd de l’Hôpital, 75013 Paris, France; 10 Michael Smith Laboratories, University of British Columbia, 2185 East Mall, Vancouver, V6T1Z4 Canada; 11 Unité de la Biologie des Cellules Dendritiques, Institut Pasteur, 25 Rue du Dr. Roux, 75015 Paris, France; 12 Centre de Recherche Translationnelle, Institut Pasteur, 28 Rue du Dr. Roux, 75015 Paris, France; 13 Centre Hospitalier Universitaire Joseph Ravoahangy Andrianavalona (CHUJRA), Antananarivo, Madagascar; 14 Centre Hospitalier Universitaire Mère Enfant de Tsaralalana, Antananarivo, Madagascar; 15 Unité d’Epidémiologie, Institut Pasteur de Bangui, Avenue de l’Indépendance, Bangui, Central African Republic; 16 Departments of Botany and Zoology, and Biodiversity Research Centre, University of British Columbia, 3200-6270 University Boulevard, Vancouver, V6T1Z4 Canada

**Keywords:** Stunting, Pediatric environmental enteropathy, Madagascar, Central African Republic, Microbiota, Immunology, Medical anthropology, Child development, Biomarkers, Risk factors

## Abstract

**Background:**

Globally one out of four children under 5 years is affected by linear growth delay (stunting). This syndrome has severe long-term *sequelae* including increased risk of illness and mortality and delayed psychomotor development. Stunting is a syndrome that is linked to poor nutrition and repeated infections. To date, the treatment of stunted children is challenging as the underlying etiology and pathophysiological mechanisms remain elusive. We hypothesize that pediatric environmental enteropathy (PEE), a chronic inflammation of the small intestine, plays a major role in the pathophysiology of stunting, failure of nutritional interventions and diminished response to oral vaccines, potentially via changes in the composition of the pro- and eukaryotic intestinal communities. The main objective of AFRIBIOTA is to describe the intestinal dysbiosis observed in the context of stunting and to link it to PEE. Secondary objectives include the identification of the broader socio-economic environment and biological and environmental risk factors for stunting and PEE as well as the testing of a set of easy-to-use candidate biomarkers for PEE. We also assess host outcomes including mucosal and systemic immunity and psychomotor development. This article describes the rationale and study protocol of the AFRIBIOTA project.

**Methods:**

AFRIBIOTA is a case-control study for stunting recruiting children in Bangui, Central African Republic and in Antananarivo, Madagascar. In each country, 460 children aged 2–5 years with no overt signs of gastrointestinal disease are recruited (260 with no growth delay, 100 moderately stunted and 100 severely stunted). We compare the intestinal microbiota composition (gastric and small intestinal aspirates; feces), the mucosal and systemic immune status and the psychomotor development of children with stunting and/or PEE compared to non-stunted controls. We also perform anthropological and epidemiological investigations of the children’s broader living conditions and assess risk factors using a standardized questionnaire.

**Discussion:**

To date, the pathophysiology and risk factors of stunting and PEE have been insufficiently investigated. AFRIBIOTA will add new insights into the pathophysiology underlying stunting and PEE and in doing so will enable implementation of new biomarkers and design of evidence-based treatment strategies for these two syndromes.

## Background

Stunting (linear growth delay) remains one of the most pressing global health problems with roughly one out of four (155 million) children under 5 years of age affected (Global Nutrition report 2017). Stunting is defined as a height-for-age z-score ≤ − 2 SD of the median height of the WHO reference population [1, 2]. In Central African Republic (CAR) and Madagascar, where AFRIBIOTA is based, the percentage of stunted children under 5 years is alarmingly high: 47% of Malagasy children [3] and 41–43% of CAR children (World Bank and Global Nutrition report, data 2010) experience stunted growth, making them two of the most affected countries in the world. Undernutrition in early childhood leads to diminished physical and mental development [4, 5], producing poor school performance and, on average, 22% less income in adulthood (Levels and Trends in Child Malnutrition, WHO, UNICEF, World Bank, 2012; [3]). Undernutrition is thus a major driver of poverty. Despite decades-long efforts to treat and reduce undernutrition through nutritional rehabilitation, these programs have been less efficacious than expected due to the persistent vicious cycle between undernutrition and infection [6, 7]. While the prevalence of stunting has slightly decreased globally in the past two decades, it has only marginally decreased in Sub-Saharan Africa, and the actual number of affected children has increased [8].

The current potential causes of stunting range from inadequate food to poor hygiene and repeated infections [6]. Stunting is a complex entity that may reflect several etiologies, particularly a poor, unbalanced diet and insufficient vitamin/micronutrient intake. It also involves social factors, including family’s resources and configuration, as well as the broader political and economic conditions in which children live [9].

To date, although evidence about social and other risk factors that contribute to stunting exists, its pathophysiological mechanisms remain largely elusive. As a consequence, there is still no proper intervention to cure stunting, and the most effective interventions correct for at best one third of the observed linear growth delay [10]. In recent years, accumulating evidence has shown that a chronic, inflammatory syndrome of the small intestine, called pediatric environmental enteropathy (PEE), may play a major role in this syndrome. [11–14]. PEE (also called tropical enteropathy or environmental enteric dysfunction) is a subclinical condition generally thought to be caused by constant fecal-oral contamination [15–19] resulting in increased permeability of the small intestine and influx of immune cells into the gut epithelium [20]. This chronic inflammation leads to characteristic shortening of the villi, diminishing the absorptive surface of the intestine (reviewed in [20–22]). It is believed that stunting and PEE are two intertwined syndromes, leading to a vicious cycle exacerbated over time [23–29]. Histopathological analysis conducted on duodenal biopsies, and microbiological studies conducted on duodenal aspirates of infants and children affected by PEE have revealed three major components supporting the current pathophysiological hypothesis [30]: intestinal atrophy through villi blunting, inflammatory infiltration into both the epithelium and the *lamina propria*, and outgrowth of pro-inflammatory *Enterobacteriae* and bona fide enteric pathogens [31]. Hence two, likely related, etiological options may explain PEE: (i) a succession of enteric infections, or (ii) a dysbiotic microbiota involving a sustained oral acquisition of fecal organisms that colonize the duodeno-jejunum, thereby creating small intestinal bacterial overgrowth (SIBO) comprised of a pro-inflammatory microbial community. This microbiota dysbiosis results in an ecosystem that cannot maintain the major parameters of gut homeostasis and function in a part of the intestine that is vital for digestion and nutrient absorption. Both scenarios might take place either in an intestine weakened by undernutrition, or might lead themselves to undernutrition, thereby initiating the vicious cycle.

The MAL-ED (Malnutrition and Early Disease) consortium addressed the first hypothesis, looking for (asymptomatic) infections leading to subsequent growth delays. They showed that intestinal inflammation and growth delay among infants in eight developing countries were associated with entero-invasive/mucosa-disrupting enteropathogens [32]. A recent study in Bangladesh concluded that enteric infections, especially of *Shigella* and enterotoxic *E. coli* (ETEC), were associated with PEE and stunting in the first 2 years of life [33]. Two other studies found entero-aggregative *E. coli* (EAEC) to be associated with markers of PEE (gut inflammation) and linear growth delay [34, 35].

The second hypothesis, stating that a dysbiosis, rather than actual infection, might lead to PEE, remains unaddressed in humans. It is nonetheless supported by several observations. Peace Corps volunteers diagnosed with EE took up to a year to recover from the syndrome, even once exposed to improved food and water hygiene upon returning to the US [36, 37]. This also implies that affected children cannot simply be fed a nutritious diet to recover from the syndrome. The observed “imprinting” stresses the significance of long-lasting effects. “Imprinting” could be mediated by specific, pro-inflammatory members of the microbiota, which remain in the microbial community even after dislocation to better hygienic conditions. Alternatively, this phenotype could be due to epigenetic imprinting, leading to changes in the general gut homeostasis. The causative role of given microbes in inducing and sustaining undernutrition is supported by two studies in mice, which reproduced the main hallmarks of PEE by chronic undernutrition and gavage with a given set of pathobionts. Further, it was possible to transfer the PEE phenotype by inoculating germ-free mice with feces of affected animals. Likewise, feces from stunted children inoculated into germ-free mice led to stunting in the recipient mice [26, 38, 39]. Human data for this second hypothesis is therefore urgently needed as to assess the pathophysiological mechanisms underlying these interactions in greater detail and to identify potential interventions.

In AFRIBIOTA, we focus on children falling under the second etiology: children with or without linear growth delay in apparently good health at the time point of inclusion. We hypothesize that stunting and PEE are caused by changes in the gut ecosystem, first and foremost the bacterial microbiota but likely also to changes induced in the pool of bile acids, the eukaryome, as well as the mucosal immune system.

PEE was described for the first time in the late 1960s, based on abnormal histology of the small intestine [40, 41]. Several other studies were performed in the following decades to explore other biomarkers of the disease [42–50]. Nevertheless, to date, characterizing PEE in the absence of biopsies showing PEE blunting and immune cell infiltration remains a challenge. Gut permeability, measured through the lactulose/lactitol-mannitol test, is the current reference test. However, gut permeability is an unspecific condition, occurring under different clinical circumstances, and with potentially diverse etiologies including infection. The test’s specificity is thus highly debated, and its value must be contextually interpreted. Furthermore, the test requires that children fast overnight, after which their urine needs to be collected over 5 h and analyzed using mass spectrometry. Therefore, this analysis remains difficult to perform in low-income settings, and expensive to conduct on a large scale.

For this reason, few PEE studies have been conducted. An easy to use, inexpensive, specific, and sensitive diagnostic test for PEE is therefore urgently needed. In the last 2 years, several studies have reported analyses of biomarkers for PEE [51, 52]. The rational of the choice of biomarkers is often based on the fact that PEE is clinically similar to inflammatory bowel disease (IBD), therefore some of the markers for IBD might also be valid for PEE [53, 54]. From the first studies performed, a general consensus emerged on the fact that (systemic) inflammation appears to be most associated with linear growth deficit [51, 52]. However, more studies are needed to validate these first results in other contexts and other age groups.

**Fig. 1:**
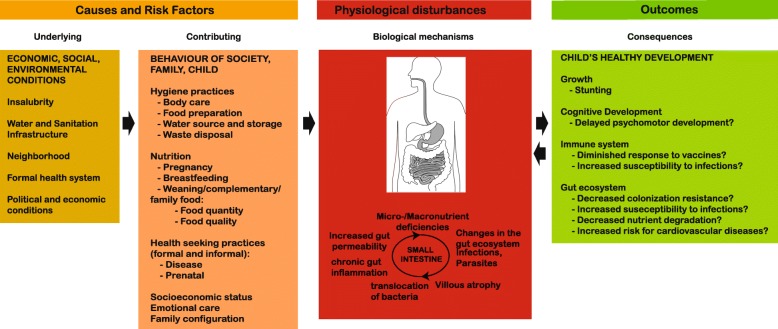
Scheme depicting the different entities underlying or being affected by pediatric environmental enteropathy (PEE). Underlying causes are colored in orange, physiological changes in red and consequences in green

In recent years, certain studies have addressed risk factors associated with PEE. The main risk factors found were nutritional status, exposure to pathogens, illness, socioeconomic status and feeding practices [32]. Further, geophagy [16] and mouthing of soil-contaminated objects [15] as well as animal exposure and caregiver hygiene [19] were also associated with an increased risk for PEE. The most important factors associated with PEE can be conceptualized by: (1) underlying and contributing factors, which are mainly of social origin; (2) biological mechanisms leading to the pathophysiological changes observed in the small intestine; and (3) pathophysiological outcomes of these small intestinal changes (Fig. 1). Many of the factors underlying PEE and stunting are tightly linked, such as parasite burden, infection, socioeconomic status and access to health care. Therefore, it is crucial to collect as much metadata as possible for each child to correct for these factors and analyze the different influences independently from each other. Only a tightly controlled study group will allow correction for a maximum of confounding factors and truly shed light on the pathophysiological associations observed upon PEE. To this purpose, in AFRIBIOTA, we decided to include a larger number of children rather than performing a longitudinal study on fewer children.

In addition to its correlation with stunting, PEE is also linked to other long-term *sequelae*, including psychomotor delay, diminished oral vaccine performance [55] and increased risk of cardiovascular diseases later in life [47]. The estimated prevalence of PEE is greater than 75% in the most affected regions [11, 13, 56, 57]. Considering its very high prevalence in low-income countries, PEE now ranks among health priorities for which efficient prevention/treatment should significantly improve childhood health and future life quality.

Several consortia have begun to investigate PEE in the previous decade [49, 58, 59]. AFRIBIOTA departs from these investigations in several ways, and is a study uniquely designed to fill in existing knowledge gaps in the field. By definition, PEE is a small intestinal disease. AFRIBIOTA collects duodenal samples that are precious for they likely contain the putative microbial biomarkers that will allow a better understanding of the ecology of affected children’s small intestines. The microbiota differs greatly between the different compartments of the gastrointestinal tract [60–62], and it is therefore important to define small intestinal microbiota present in the context of PEE and stunting. Secondly, different candidate biomarkers are simultaneously measured in a group of almost 1000 children, to better delineate the components of PEE. This will allow a comparison of the different markers and to develop models to design a multi-parametric composite test to discriminate PEE from other gastrointestinal disorders. Finally, AFRIBIOTA combines different disciplines and approaches to understand the conditions facilitating and sustaining PEE and growth delay. These approaches will yield detailed evidence concerning each child, allowing screening for associations between social and biological factors.

The main objective of the AFRIBIOTA project is to shed light on the interactions between dysbiosis and stunting/PEE in children between the age of two and 5 years.

Secondary objectives include i) testing a panel of candidate biomarkers for PEE, ii) investigating the broader social environment and epidemiological risk factors for stunting and PEE and iii) describing possible associated pathophysiological changes in the mucosal and systemic immune system as well as delayed psychomotor development in children.

In conclusion, AFRBIOTA promises to add valuable insight to the developing picture of the pathophysiology underlying stunting and PEE, and to extend existing efforts to comprehend these two syndromes.

## Methods/Design

### General study design/recruitment

AFRIBIOTA is a matched case-control study for stunting. In order to correct for study-site specific variables (ex: climatic factors, food habits, overall genetic make-up of the population), we opted to perform the study in two distinct study countries (Madagascar and Central African Republic). Three different categories of children aged 2–5 years are enrolled in the study: severely and moderately stunted children (100 of each group/ country) and children with no growth delay (260/country). Severe stunting is defined as a height-for-age z-score ≤ -3SD, moderate stunting as height-for-age z-score between -3SD and -2SD of the median height of the WHO reference population [1, 2]. Control children are children without stunting (height-for-age z-score > 2SD). Stunted and control children are matched according to age (24–35 months, 36–47 months and 48–60 months), gender and neighborhood (same neighborhood or adjacent neighborhood as based on the official maps distributed by the respective Ministries) and season of inclusion (dry or wet season). As PEE cannot be measured in the field with the diagnostic tests currently available, we hypothesized that most stunted children display PEE while most of the non-stunted children would display the syndrome at a lower level of severity or not at all. Stunting was therefore taken as a proxy for PEE. Recruitment started in December 2016 in Antananarivo and in January 2017 in Bangui and is currently ongoing. Recruitment should be completed by summer 2018. The study includes a total of 920 children.

### Inclusion and non-inclusion criteria

We applied the following inclusion and exclusion criteria: i) children being between 24 and 60 months old and capable of participating in the different tests and clinical sampling; ii) not showing any of the following exclusion criteria: severe acute illness, acute malnutrition or enteropathy, including HIV-associated enteropathy or severe diarrhea and iii) not under recent antibiotic treatment or renutrition regimens (to avoid bias in composition of the dysbiosis associated with stunting and/or PEE, as both of these interventions were shown to lead to severe changes in the microbiota composition [63–67]) (Table 1).

**Table 1:** Inclusion and exclusion criteria

Inclusion criteria	Exclusion criteria
ᅟ•ᅟChildren between the age of 24 and 60 months ᅟ•ᅟGeneral health status allowing for the tests to be performed	•ᅟHIV positive test at inclusion •ᅟSigns of respiratory distress (≥40/min) •ᅟFever (≥ 38.5 °C) •ᅟInfectious diarrhoea with mucus or blood •ᅟAntibiotics taken in the 2 weeks prior to inclusion •ᅟRenutrition regime taken in the 6 months prior to inclusion •ᅟSeptic shock •ᅟVomiting •ᅟAcute malnutrition (WHZ ≤ − 2)

### Recruitment procedures

#### Madagascar

In Antananarivo, the recruitment is community- (90%) and hospital-based (10%). We expected challenges with acceptance by the parents on performing aspirations on awake children. Therefore, hospitalized children were included to facilitate duodenal aspirations, as they could be performed during surgical interventions when the child is under narcotics. Nevertheless, so far, all aspirations were performed on awake children and, thanks to a detailed and complete information package delivered by the caregivers, no issues arose concerning acceptability of the procedure.

Community recruitment is performed in Ankasina and Andranomanalina Isotry, two of the poorest neighborhoods of Antananarivo, as well as their surrounding neighborhoods. Families are informed about the study by community health workers and sent to a weekly recruitment event at the community health center of the respective neighborhood where they are measured, inclusion and exclusion criteria checked, and appointments are scheduled for the different tests (community-recruited children). Children who seek care in the Centre Hospitalo-Universitaire Mère Enfant de Tsaralalàna (CHUMET), in the Centre Hospitalo-Universitaire Joseph Ravoahangy Andrianavalona (CHU-JRA) and in the Centre de Santé Maternelle et Infantile de Tsaralalana (CSMI) and meet the inclusion and exclusion criteria are also invited to participate in the study (hospital-recruited children).

#### CAR

**Fig. 2:**
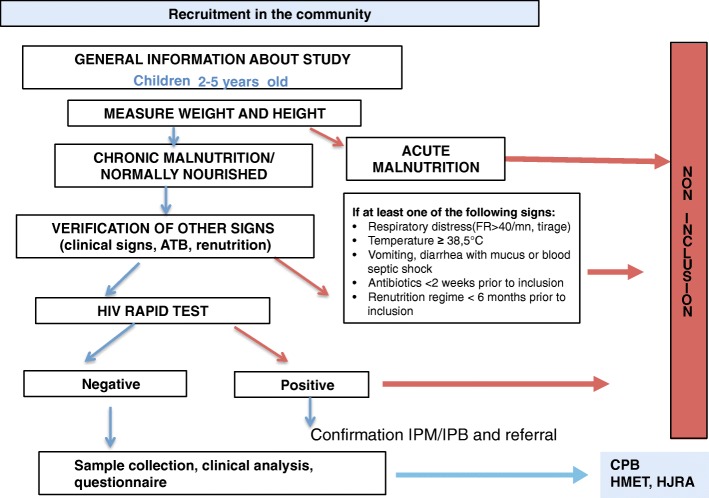
Recruitment Schema of the AFRIBIOTA project

In Bangui, all recruitments are conducted in the community. Children are recruited in three districts (6th, 7th and 8th arrondissement), randomly selected among the 14 districts of Bangui. Community health workers approach families, inform them about the study, and send them for inclusion to the arrondissement health center, where recruitment sessions take place every 3 weeks (Fig. 2).

### Variables collected

#### Anthropometric measurements

Height is measured to the nearest 0.1 cm in a standing position using collapsible height boards (Antananarivo: ShorrBoard^®^ Infant/Child/Adult Measuring Board, Maryland, USA; Bangui: height board provided by UNICEF); weight is measured to the nearest 100 g using a weighing scale (Antananarivo: KERN, ref. MGB 150 K100, Antananarivo, Madagascar and EKS, Inter-équipement Madagascar; Bangui: weighting scale provided by UNICEF). Head circumference is measured around the widest possible circumference to the nearest 0.1 cm using a flexible measuring rod. Mid-upper arm circumference (MUAC) is measured using commercial MUAC tape (provided by UNICEF) as follows: first, the tip of the shoulder and the tip of the elbow are determined and distance is measured. The mid-point between these two points is marked and the MUAC tape is applied. Arm circumference is measured to the nearest 0.1 cm.

#### Biological measurements and tests performed

**Fig. 3:**
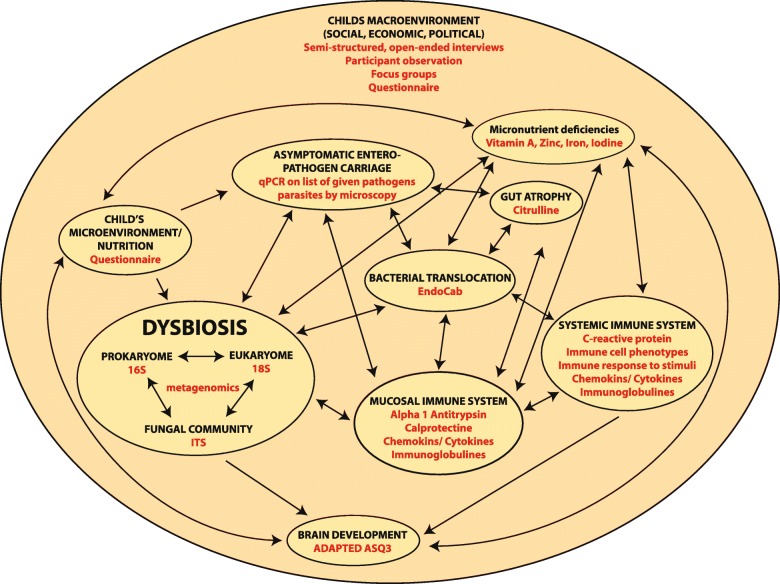
Framework of the different interacting entities being associated with stunting and pediatric environmental enteropathy (PEE). Data collected for each entity in the context of the AFRIBIOTA project is indicated in red. Interactions in between the different entities are indicated with arrows. The child’s macro-environment is influencing all other entities

We measure different interacting entities that might play a role in the pathophysiology of child stunting and PEE (Fig. 3). They include the pro- and eukaryotic microbial community in the small intestine as well as in gastric aspirates and feces; gut atrophy; the mucosal and systemic immune response; micronutrient deficiencies; asymptomatic enteropathogens and parasite carriage; gut leakiness and atrophy and bacterial translocation; and the micro- and macro-environment of the child. For each child, feces, urine and blood are collected. For stunted children (200 children/country), we also collect gastric and duodenal aspirates. We apply both culture techniques and NGS (16S, 18S, ITS amplicon sequencing, metagenomics) to determine the community structure of the small intestinal aspirates, hence generating unprecedented data about the small intestinal community structure in children living in low-income countries. We also assess the microbial composition of feces using NGS and investigate the IgA-targeted fraction of the microbiota as to have a detailed picture of the immunogenic bacteria. Furthermore, we assess for asymptomatic pathogen carriage using qPCR targeted against the most prevalent enteropathogens and assess for the presence of parasites using conventional microscopy techniques (direct examination, Kato-Katz and MIF).

To analyze the gut ecosystem in more detail, we also describe the pool of bile acids in the duodenum and feces using targeted mass-spectrometry, describe the pool of cytokines and chemokines using a commercially available panel of 30 cytokines/chemokines/growth factors (Invitrogen; Luminex MagPix technology) as well as a commercial Luminex Assay against the different subtypes of Immunoglobulins (Biorad). The systemic immune system is analyzed using 8-color flow cytometry and a panel of pre-established antibody sets [68] to quantify the different immune cell populations, the same 30-plex kit for cytokines/chemokines/growth factors also used on intestinal samples as well as an in vitro stimulation system to assess for immune responses against given stimuli, the TruCulture technique [69] (see Table 3 for a detailed description of the different aspects addressed).

We integrate changes in the microbiota (bacteria, eukaryotes, asymptomatic pathogen carriage) and the bile acid pool and correlate it with changes in the mucosal and peripheral immune system. Further, we analyze if the permeability of the gut in these children leads to translocation of bacteria into the bloodstream and could therefore lead to the chronic inflammation observed.

#### Developing a better diagnostic test for pediatric environmental enteropathy

The current gold standard diagnostic test for PEE, the lactitol-mannitol test, requires resources and technical knowledge that are frequently unavailable in resource-poor settings. Hence, to date, only limited epidemiological surveys could be performed.

**Fig. 4:**
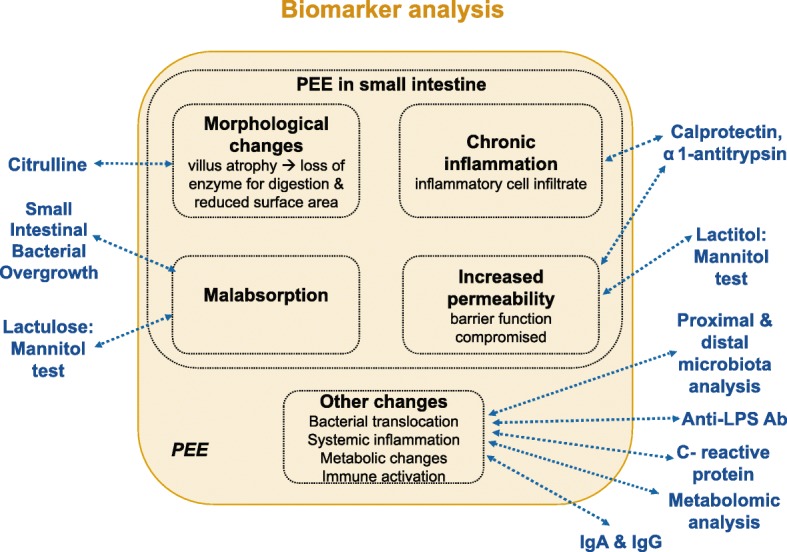
Schema of the biomarker analysis performed in AFRIBIOTA. Features assessed are indicated in black, measurements performed within the context of AFRIBIOTA in blue

In order to identify novel biomarkers and compare different tests with the reference test for PEE, the lactitol-mannitol test, a set of nine different candidate biomarkers/biomarker groups reflecting different aspects of the syndrome will be analyzed. The candidate biomarkers describe i) gut permeability, ii) mucosal inflammation, iii) systemic inflammation, iv) activation of the adaptive immune system (as reflected by the production of immunoglobulins), v) gut atrophy, vi) bacterial translocation vii) small intestinal bacterial overgrowth (SIBO), viii) specific taxa of the fecal microbiota (including pathogenic or non pathogenic bacteria, viruses and eukaryotes) and ix) specific bile acid profiles. The choice of the respective candidate biomarkers is summarized in Fig. 4 and Table 2 and detailed below.

**Table 2:** Candidate biomarkers for environmental enteropathy

Candidate biomarkers	Pathophysiological change measured	Sample type needed for analysis
Lactitol-mannitol test	Intestinal permeability	Urine
Citrulline	Villous atrophy	Blood
α anti-trypsin	Altered intestinal barrier	Faeces
Calprotectin	Mucosal inflammation	Faeces
C reactive protein (CRP)	Systemic inflammation	Blood
Endotoxine (circulating LPS)	Bacterial leakage into the systemic circuit (intestinal permeability)	Faeces
Immunoglobulines	Adaptive immune response	Blood, faeces, duodenal aspirates
Small intestinal bacterial overgrowth	Too important bacterial load in the small intestine	Duodenal aspirates
Specific bacteria or eukaryotes	Disturbances in the gut ecosystem	Faeces, duodenal and gastric aspirates
Specific bile acid profiles	Disturbances in the gut ecosystem	Faeces, duodenal and gastric aspirates

##### Gut permeability

Gut permeability is measured using the lactitol-mannitol test [70]. Lactitol and mannitol are both non-metabolized sugars and are secreted unchanged in the urine after absorption. Their levels reflect the permeability and the absorptive capacity of the intestine, respectively. It is the current reference test for PEE [11, 32, 52, 71, 72].

##### Systemic inflammation

The presence of a systemic inflammatory response is analyzed by blood dosage of the inflammatory marker C-reactive protein (CRP). CRP is an acute-phase protein that is quickly up-regulated and expressed in response to a variety of viral, bacterial and fungal infections as well as other non-infectious inflammatory states.

##### Local inflammatory response in the gut

The local inflammatory response in feces (all children) and in gastric and duodenal aspirates (stunted children only) is analyzed by dosing two different inflammatory markers: alpha1-antitrypsin and calprotectin. Alpha1-antitrypsin is released during an inflammatory state by leucocytes. Calprotectin is secreted by neutrophils and has been shown previously to be a valid biomarker for intestinal inflammation, for example in the context of inflammatory bowel disease [54]. Both markers have also been used as biomarkers in recent studies on PEE [52] and have been shown to be associated with subsequent linear growth delay.

##### Immunoglobulin levels (adaptive immune response)

An important quantity of immunoglobulins (Ig), especially IgA, but also IgM is secreted every day in the intestinal lumen where it contributes to regulate the microbiota. An imbalance in the levels of Igs can lead to dysbiosis (reviewed in [73]). Bacterial overgrowth can also enhance secretion of IgG into the gut lumen (reviewed in [41]) and can induce a higher secretion of IgG into the blood, reflecting to some extent the permeability of the intestine. It has been previously described that in the context of undernutrition, IgG concentrations in the blood are enhanced while IgA concentrations in the gut (feces) are diminished [74]. It has also been described that Vitamin A and Vitamin D deficiencies lead to a diminished production of IgA in response to different viruses (reviewed in [43]). PEE leads at the same time to undernutrition and malabsorption resulting in diminished levels of different vitamins and trace elements [75]. We therefore hypothesize that the general levels of immunoglobulins might be affected by PEE.

##### Intestinal atrophy (villous abrasion)

The mass of enterocytes, hence the degree of intestinal atrophy, can be measured by dosage of blood citrulline as this amino acid is only secreted by enterocytes. Indeed, seric citrulline levels were able to correctly predict the enterocyte mass in the context of HIV-induced enteropathy [76]. Citrulline is therefore a good substitute for histological scoring of villous length in biopsies [76–78]. In a recent study, citrulline levels have also been shown to be associated with subsequent linear growth delay [52]. Since PEE is characterized by villous atrophy, citrulline levels may be an easy to measure, reliable marker of PEE.

##### Bacterial translocation (circulating LPS)

Several studies have assessed circulating LPS as a biomarker for PEE. While some studies showed a clear association between circulating anti-LPS antibodies and gut leakiness/linear growth delay [46, 52, 79, 80] others did not [81]. Bacterial translocation being a plausible outcome of the pathophysiology described so far for PEE, we include the endotoxin core antibody (EndoCAb® test, Hycult Biotech, Uden, The Netherlands) in the panel of biomarkers to be tested.

##### Small Intestinal bacterial overgrowth (SIBO)

PEE and undernutrition have been associated with small intestinal bacterial overgrowth [50, 82, 83]. SIBO might therefore be a good biomarker for PEE. SIBO can be measured either by the hydrogen breath test [84, 85] or by direct plating of duodenal aspirates [86]. In AFRIBIOTA, we opted to use the plating method to simultaneously evaluate both the presence of SIBO, and its microbial composition.

##### Fecal bacterial taxa associated with PEE

It was shown recently in a pilot study that PEE is accompanied by changes in the fecal microbiota [48]. Further, the fecal microbiota of children with SIBO was also shown to be different [87, 88]. It is therefore likely that given taxa could be valid biomarkers for PEE. Taxa that show significant association with PEE in a multivariate model, will be assessed for their potential use as biomarkers.

##### Specific bile acid profiles

Bile acids are important in lipid absorption and they also play a major role in shaping the microbiota. In turn, the microbiota converts primary bile acids into secondary bile acids by chemical reactions including dehydroxylation, epimerization, oxidation, esterification, and desulfatation, among others [89]. In a recent study, bile acid profiles in the blood were changed in the context of PEE with overall lower amounts of serum bile acids and a higher proportion of bile acids conjugated with taurine in PEE children compared to their non-PEE controls [90]. It is therefore likely that in the context of PEE fecal bile acid pools are changed as well and might represent a good biomarker for the disease.

#### Understanding the broader environment of children with PEE

In both countries, a medical anthropological study is conducted in the recruitment districts to evaluate children’s social and environmental living conditions. Using an observation grid, we observe hygienic practices, including hand washing, food preparation and consumption, and water storage. Further, data about the medical history of the child, the mother’s pregnancy, child feeding and care practices as well as household characteristics are collected with a standardized questionnaire. The data of these questionnaires will be brought into dialogue with household-level participant-observations. We will use additional medical anthropological methods to produce analyses (semi-structured, open-ended interviews, focus groups, …) of the children’s social relations (with caregivers, other family and neighbors) and economic and environmental conditions (Table 3). This evaluation will contribute to the development of socially appropriate intervention and prevention strategies and tools. Comparing data on risk factors associated with PEE from our two study sites, as well as with published data from other studies, will enable us to generalize the observed risk factors and identify possible interventions to minimize the risk of developing PEE.

**Table 3:** Aspects of PEE and stunting studied by the AFRIBIOTA study group

*Aspect addressed*	*Reasoning*	*Methods used*	*Study site*	*Statistical considerations*
Social relations, political and economic conditions of children	Stunting and PEE are linked to poverty. Specific political economic conditions and social relations appear to be drivers of these two syndromes.	Participant-observations Open-ended interviews focusing on life histories, family histories, specific practices of social interactions, hygiene and feeding of children GPS mapping of major points in neighborhoods (food, contamination, play areas, waste disposal, etc.)	Bangui & Antananarivo	30 families with a stunted child/ child with PEE and 30 families with a non-stunted child per country or until exhaustion. Data analysis using “grounded theory” approach
Risk factors	To date, very little is known about the actual risk factors for PEE, a fact that hampers developing evidence-based prevention strategies.	Standardized questionnaire about the general health status of children, nutrition, family composition, hygiene and mother’s pregnancy Biological data on micronutrient deficiencies, inflammation, parasite load	Bangui & Antananarivo	Hypothesizing a PEE prevalence of 75% in controls [57, 80] and 85% in cases, to show an odds ratio of 4.8 a power of 80% and a two-sided α = 0.05, an expected proportion of exposed controls of 32%, a sample size of 30 stunted children and 100 non-stunted controls is needed. Accounting for 10% of secondary exclusion, the required sample size is 34 stunted children and 111 non-stunted control children.
Diagnostic test	To date, the reference test for PEE, the lactitol-mannitol gut permeability test, is difficult and costly to perform in low-income settings. Further, gut permeability is a non-specific aspect of any inflammatory disease of the intestine. Efforts are therefore needed as to find other, more specific and easier to use biomarkers of the syndrome. The lactitol-mannitol gut permeability test is therefore an imperfect test.	Measurement of a given set of nine different biomarkers Sensitivity/Specificity testing against the reference test and latent model of the different markers.	Bangui & Antananarivo	Sample size was calculated based on the formula provided by Beam et al. [92] for matched-groups diagnostic study. Assuming a sensitivity and specificity of 80% for the candidate biomarker, respectively (the sensitivity and specificity of the imperfect reference test were estimated to be respectively 90 and 80%), with a power of 80%, a probability of disagreement of 0.18 between the two test, an assumed secondary exclusion of 10%, the total estimated sample size is of 128 children, 64 with PEE and 64 without PEE. With an estimated PEE prevalence of 85% among the stunted children and 75% among the non-stunted controls [80], 75 stunted children and 256 non-stunted controls have to be included, hence a total of 331 children.
Asymptomatic enteropathogen carriage	It is well established that diarrhea and undernutrition complement each other in a deleterious vicious circle, however, the prevalence of PEE seems higher among the pediatric population than the prevalence of recurrent/chronic diarrhea [11, 80]. The degree of overlap between these two entities remains unclear. To date, data on possible links between asymptomatic pathogen carriage and stunting remain scarce. The MAL-ED consortium found a relation between asymptomatic enteropathogen carriage and stunting, which likely is mediated through systemic inflammation [100]. It is likely that other pathogens might also contribute to PEE in subclinical infections.	qPCR on a given list of enteric pathogens (bacteria, viruses and parasites) Microscopy for parasites	Bangui & Antananarivo	Based on earlier studies in Antananarivo [103] and Bangui [104, 105] we assume a prevalence of roughly 10% of any asymptomatic microorganism carriage, among children. To show an odds ratio of 3 between stunting and any asymptomatic pathogen carriage, with an asymptomatic pathogen carriage prevalence of 10% in non- stunted children [105], a power of 80% and a two-sided α = 0.05, a sample size of 97 stunted children and 97 non-stunted controls are to be recruited. To show an odds ratio of 3 between the carriage of a given parasite and PEE, with a pathogen prevalence of 10% in non-PEE children, a power of 80% and a two-sided α = 0.05, a sample size of 97 PEE children and 97 non-PEE controls are to be recruited. Assuming a PEE prevalence of 85% among the stunted children and 75% among the non-stunted controls [80], 115 stunted children and 380 non-stunted controls are to be recruited to assess the association between nutritional status and PEE. With a secondary exclusion of 10%, this sums to a total of 475 non-stunted children and 144 stunted children for each country. The analysis will be performed pooled on both countries as to have enough sample size.
Small intestinal bacterial overgrowth (SIBO)	Impaired small intestinal barrier functions – possibly also impaired digestive and nutrient transport functions - seem to be largely caused by the stable constitution of small intestinal bacterial overgrowth (SIBO) [50, 106] causing local and systemic endotoxemia, thus excessive local and systemic inflammation [83]. SIBO is prevalent in shanty towns in many places [50, 83, 107, 108] and it is therefore likely that SIBO might have a role in PEE.	Culture of duodenal samples (SIBO > 10^5^ bacteria/ml of aspirate)	Bangui & Antananarivo	SIBO analysis can, for ethical reasons, only be performed on stunted children. In the context of this study, 400 stunted children (200 severely stunted and 200 moderately stunted) will be recruited and aspirated. We therefore could not estimate a sample size, but will analyze all collected samples and compare the moderately to the severely malnourished children estimating the power retrospectively (exploratory analysis):
Microbial composition of the gastrointestinal tract (primary objective)	It has long been speculated that the microbiota might be changed in PEE. However, to date, only a single study in fecal samples was performed, showing changes in the gut microbiota of PEE children compared to their healthy controls [48]. One of the strengths of AFRIBIOTA is its capacity to collect samples in their most relevant location, particularly the collection of duodenal fluid in affected children, which will allow studying the microbiota composition at the location where disease takes place.	Amplicon sequencing (16S, 18S, ITS) IgA targeted bacterial fraction (BugFacs) Metagenomics	Bangui & Antananarivo	We estimate at least 100 samples per categories and per country are required (effect size unknown, convenience sampling).
Bile salt profiles	Primary bile acids are crucial players in fat absorption. They are transformed into secondary bile acids by the resident gut microbiota. Bile acids are shaping the microbiota by promoting the growth of bile acid-metabolizing bacteria and by inhibiting the growth of bile-sensitive bacteria. In a recent study, serum bile acid profiles were changed in PEE children [90].	Mass spectrometry analysis	Bangui & Antananarivo	We estimate at least 100 samples per categories and per country are required (effect size unknown, convenience sampling).
Mucosal immune system	To date, while it is increasingly clear that mucosal immune dysfunction is linked to stunted growth [109], there is little knowledge on how PEE affects the immune system of the small intestine. Campbell et al. showed that PEE leads to an increased presence of T-cells in the *lamina propria* and the epithelium of the small intestine of children with PEE [45, 46]. Brown et al. showed that in a weaned mouse model for PEE, the presence of NK T cells in the gut was increased [26]. In humans, it is highly challenging to analyze the mucosal immune system. As specific immune cells release specific cytokines, we will assess the presence or absence of individual cytokines.	Cytokine/Chemokines/Growth Hormone profiling Immunoglobulin profiling	Bangui & Antananarivo	We estimate at least 100 samples per categories and per country are required (effect size unknown, convenience sampling).
Systemic immune system	In the context of chronic enteropathy, the ratio of circulating T_H_17 to T_reg_ cells is increased [109, 110]. To date, nothing is known about the circulating cell populations in PEE. We therefore analyze the circulating cell populations of children with chronic undernutrition and/or PEE. We will use five eight-color antibody panels as well as cytokine and immunoglobulin profiling in order to quantify and characterize the major leukocyte populations and their secreted immune molecules.	Cytokine/Chemokines/Growth hormone profiling Immunoglobulin profiling Flow cytometry (B-cells, NK-cells, monocytes and the different subsets of CD4+ T-cells (T_H_1, T_H_2, T_H_17 and T_reg_) [68].	Bangui (cytokine/immunoglobulin profiling) & Antananarivo (cytokine/immunoglobulin profiling and flow cytometry)	We estimate at least 100 samples per categories and per country are required (effect size unknown, convenience sampling).
Mounting of immune responses	Vaccines are performing less well in the developing world than in industrialized countries [59]. We hypothesize that this is due to changes in immune homeostasis and abrogated immune responses as a result of PEE, an association that was previously shown in a few studies [55, 93, 110, 111]. In low-income settings, there is widespread absence of trustworthy vaccine records. Therefore, we aim at using an alternative approach, the TruCulture stimulation system (Myriad). This allows investigating the immune response of children in vitro [69]. Stimuli selected include LPS (gram negative bacteria), Poly I:C (double stranded RNA viruses) and staphylococcal enterotoxin B (SEB) (T-cells). These stimuli were selected as they represent the two groups of pathogens that cause the most orally contracted infections in small children and target the T-cell response implicated in PEE pathophysiology [26, 46].	TruCulture system (Myriad)/ Cytokine/Chemokines/Growth hormone profiling [69]	Antananarivo	We estimate at least 100 samples per categories and per country are required (effect size unknown, convenience sampling).
Psychomotor development of children	Changes in the microbiota and its metabolites have been associated since several years with brain development (“gut-brain axis”) [112]. Recent reports also reported helminth carriage and associated microbiota changes as one of the risk factors of delayed psychomotor development [113]. Considering the role these entities play in PEE, it is therefore likely that PEE is associated with psychomotor delays [114].	Adapted version of the ASQ3 test	Antananarivo	For the psychometric analysis internal consistency (Cronbach’s alpha), test-retest, and inter-rater (Kappa statistics with expert child development specialist) are performed. Validity of the tool is measured using the Pearson product moment test and factor analysis (FA). For association with PEE, we estimate to required at least 100 samples per category (effect size unknown)

#### Psychomotor development

Each child included in Madagascar undergoes a psychomotor development evaluation with a specifically adapted version of the Ages and Stages 3 Questionnaire (ASQ3, Ages & Stages Questionnaires®, *Third Edition* (ASQ-3™), Brookes Publishing Co), capturing a child’s development in five different spheres (fine motor, gross motor, communication, problem solving, and personal-social). Trained, local psychologists perform the evaluation. The questionnaire was translated to Malagasy and all items were culturally adapted to the local context. We will correlate gut permeability, local and systemic immune status as well as given microbiota profiles with psychomotor development to find biological patterns that might be associated with the psychomotor delay observed in stunted children/ children suffering of PEE.

### Quality control and validation

The recruitment procedures and laboratory protocols were tested before the start of the full study in a pilot study in Antananarivo, Madagascar (15 children included). Procedures were subsequently reviewed and adapted for the full study. An important component of the Afribiota consortium lies in strengthening the research capacities of younger scientists and of the participating African centers, including the different hospitals. Training sessions for good clinical practices, the different medical procedures performed, anthropological and child development techniques as well as of the laboratory techniques were performed in Madagascar and in the Central African Republic prior to the activities. Data management is harmonized between the two study sites and quality control missions took place in both study sites at regular intervals to assess harmonization of laboratory and clinical protocols and control quality of the activities performed. Data is entered in double and controlled by an external data manager.

### Statistical considerations

#### Sample size

To answer all primary and secondary objectives the sample size is of 460 children per country (100 moderately and 100 severely stunted children and 260 non-stunted controls).

For the primary objective, the assessement of the gut ecosystem in the context of stunting and PEE, based on earlier microbiota studies of stunting [91], we estimate to need at least 100 samples per category (non-stunted, moderately or severely stunted) and per country (convenience sampling). Secondary objectives of AFRIBIOTA include the validation of candidate biomarkers for PEE. Sample size for this secondary objective was calculated based on the formula provided by Beam et al. [92] for matched-groups diagnostic study. Assuming a sensitivity and specificity of 80% for the candidate biomarker, respectively (the sensitivity and specificity of the imperfect reference test were estimated to be respectively 90 and 80%), with a power of 80% a probability of disagreement of 0.18 between the two tests, an assumed secondary exclusion of 10%, the total estimated sample size is of 128 children, 64 with PEE and 64 without PEE. With an estimated PEE prevalence of 85% among the stunted children and 75% among the non-stunted controls [80], 75 stunted children and 256 non-stunted controls have to be included, hence a total of 331 children.). This sample size will also allow identifying risk factors associated with stunting and PEE with an odds ratio of at least 4.8 as well as performing all other secondary objectives (see Table 3 for a detailed description of the statistical calculation of the different planned analyses).

#### Definition of PEE

Initially, gut permeability (lactitol-manitol test) will be used as reference test, as it has been widely used in the literature for defining the syndrome [80]. Each marker will then be compared to the reference test. A second analysis will be performed without using the reference test, considering that all markers have the same weight (latent class analysis model). From these results a reference composite score will be elaborated. PEE will be defined throughout AFRIBIOTA using the lactitol-mannitol value as well as the new composite score.

## Discussion

Despite its broad recognition as a major global health problem, we still know little about the pathogenic mechanisms associated with PEE. It is unlikely that any significant progress will be made towards controlling this syndrome without a clearer understanding of the molecular mechanisms underlying it.

PEE has been studied in the last few years by several groups, including the MAL-ED [49], PROVIDE [93] and SHINE [58] consortia, focusing mainly on very young children (“first 1,000 days”). The first 1000 days have been associated with the most dramatic effect on linear growth delay [94]. Given the complex interactions taking place in undernutrition it is crucial to analyze the syndrome in different countries and in different age groups in order to generalize observations. In AFRIBIOTA, the age range of children included is outside the so-called “first 1000 days”. There are several reasons for this: firstly, composition is highly dynamic and diverse in the first 2 years of life ([95], reviewed in [96, 97]), making it difficult to generalize observed phenotypes in a cross-sectional study. At 2–3 years of age, the microbiota stabilizes and hence makes integration with pathophysiological changes more robust. Secondly, while most of the linear growth delay was indeed shown to be acquired in the first 2 years of live, several organs and physiological functions still develop after this initial period, notably the immune system, cognitive functions and several important organs [98]. Chronic undernutrition and stunting beyond the first 1000 days still has a major effect on healthy child growth and linear and psychomotor catch-up growth is to some extent still possible [99]. Studying and treating PEE in children after age 2 therefore is likewise of concern to assure healthy growth. Further, there are practical and ethical issues involved which constrained our study to only include children > 2 years of age. Older and hence taller children allow also collecting a larger volume of blood, which allows testing more factors simultaneously.

The MAL-ED consortium was able to shed some light on the pathophysiological mechanisms underlying PEE in children aged 0–2 years [48, 100] and the same consortium as well as the PROVIDE consortium also started comparing and validating other biomarkers for PEE than the actual reference test, the lactitol-mannitol test [52, 101]. With PEE being so poorly characterized, it is likely that different sub-forms of the disease exist, some leading to bacterial translocation and systemic inflammation, while others lead only to local inflammation in the gut mucosa. The etiologies underlying the syndrome might also vary according to a child’s age, nutritional status or living environment. It is therefore crucial to study PEE in different settings in order to capture the complexity of the disease. Furthermore, additional insights are needed into the pathophysiological mechanisms of this syndrome.

AFRIBIOTA, to the best of our knowledge, is the first study aimed at describing the microbiota of stunted children and children with PEE in its most relevant place: the small intestine. This makes it a very valuable addition to the existing studies. Another asset of the study is its geographical location, describing phenotypes in children where, thus far, no studies on the microbiota of healthy or diseased children have been performed. This will allow a comparison of the results of the two countries with each other as well as with other published studies, hence delineating pathophysiological mechanisms and risk factors which are either conserved between different study sites, or which might be specific for a given region (“regional pathophysiological changes”).

AFRIBIOTA does have some limitations- importantly, it is a cross-sectional rather than a longitudinal study. This choice, mainly due to financial constraints, will only allow establishing associations but not causality. Nevertheless, we will include almost 1000 children from two distinct countries and will be able to integrate many different aspects of the syndrome in each child. Further, AFRIBIOTA will also generate basic microbiological data and a biobank, which will allow experimental validation of causality in animal models. We hypothesize that, in stunting and PEE, the whole microbial community rather than just isolated members might contribute to morbidity (“ecological Koch’s postulates”, reviewed in [102]). Therefore, causality can only be experimentally proven by using human samples. The causative role of dysbiotic communities on health can be tested by transplanting these communities into germfree animals, hence isolating the effect of the dysbiosis from other potentially cofounding factors, including social or environmental risk factors. The biobank established in the context of AFRIBIOTA will therefore open the way for targeted, mechanistic animal studies on stunting and PEE.

Through its interdisciplinary nature, AFRIBIOTA has the potential to profoundly change our knowledge about the intestinal ecology of children affected by stunting and PEE as well as the social and biological causes and consequences of these two syndromes. This will help to establish relevant prevention and interventions strategies, for example by targeting a specific behavior or by using probiotics or specific metabolites to treat the syndrome. Ultimately, improved prevention and treatment of PEE is essential to the growth and development of young children around the world.

## Abbreviations

ASQ: Ages-and-stages questionnaire
CAR: Central African Republic
CCTIRS: Comité consultatif sur le traitement de l’information en matière de recherche dans le domaine de la santé
CNIL: Commission Nationale de l’Informatique et des Liberté
CPB: Centre Pédiatrique de Bangui
CRF: Case report form (cahiers d’observation)
CRP: C reactive protein
CSMI: Centre de Santé Maternelle et Infantile de Tsaralalàna
CSP: Code de Santé Publique
EAEC: Enteroaggregative *E. coli* (*E. coli* enteroaggregatif)
EIGI: Effet Indésirable Grave Inattendu
EPEC: Enteropathogenic *E. coli* (*E. coli* enteropathogène)
ETEC: Enterotoxic *E. coli* (*E. coli* enterotoxique)
FACS: Fluorescence-assisted cell sorting
HJRA: Hôpital Joseph Ravoahangy Andrianavalona
HMET: Hôpital Mère Enfant de Tsaralalàna
Ig: Immunoglobulin
MUAC: Middle-upper arm circumference
PEE: Pediatric environmental enteropathy
qPCR: Quantitative polymerase chain reaction
SIBO: Small intestinal bacterial overgrowth
sp.: Species
UNICEF: United Nations International Children’s Emergency Fund
WAZ: Weight-for-Age z-score
WHO: World Health Organization
WHZ: Weight-for-Height z-score
